# Asylums in India

**Published:** 1893-02-04

**Authors:** 


### Asylums in India.

### I.—The Bombay Presidency.

There are six lunatic asylums in this Presidency, situated at Colába, Ratnágiri, Poona, Dhárwár, Ahmedabad, and Hyderabad, and the total number of their inmates in 1891 was 961, of whom 690 remained from the previous year, while 252 were admitted and 19 were re-admitted during 1891. There were 182 discharges, 90 of whom were cured and 90 transferred to friends either improved or not improved, and two escaped; the deaths numbered 70, so that at the end of the year 709 patients remained under treatment. The daily average strength was 713 3, against 706·1 in 1890. The percentages of cures and deaths to the daily average strength were 12·6 and 9 8 respectively, while the percentages to admissions were 33·2 and 25 8.

With regard to criminal lunatics, the daily average strength of this class of insane was 104, as compared with 103 in 1890. None of this class are admitted into the Ratnágiri and Dhárwár Asylums.

The general returns as to the causes of insanity offer nothing of special interest, but with regard to the causes leading to insanity, confined for crimes of violence, it is noteworthy that ganja smoking ranks first, spirit drinking taking second place, while only one case is attributed directly to opium-eating.

The financial statements show that the total expenditure was Rs.127,424 15 10, against Rs.129,172 4 11 in 1890. The receipts from paying patients amounted to Rs.12,746, and Rs.1,919 were remitted to the Treasury from the sale of manufactured articles. Deducting the above receipts from the sum of the amounts received from the Treasury, adjusted to Government, and value of articles received from the Manufacturing Department made over for the asylum purposes, the net cost to Government was Rs.97,978 13 6, as compared with Rs.93,010 10 7 in 1890. The increase is attributed mainly to the small receipt from paying patients and a large increase in the charges on account of works done by the Public Works Department in all the asylums except at Ahmedabad. The cost per patient was Rs.137 5 8, against Rs.131 11 6 in 1890.

The following table shows the daily average strength in each asylum, the net cost, and the cost per head for the last two years:—

| | Daily Averg. Strngth in 1891. | Net Cost. | | | Cost per Head. 1891. | | | 1890. | | |
|---|---|---|---|---|---|---|---|---|---|---|
| | | Rs. | a. | p. | Rs. | a. | p. | Rs. | a. | p. |
| Colába ........ | 280·7 | 55,417 | 8 | 6 | 197 | 6 | 9 | 194 | 6 | 8 |
| Ratnágiri ...... | 69·2 | 8,740 | 5 | 2 | 126 | 4 | 9 | 132 | 15 | 8 |
| Poona.......... | 97·0 | 10,479 | 9 | 10 | 108 | 0 | 7 | 105 | 6 | 11 |
| Dhárwár ...... | 38·0 | 4,566 | 4 | 10 | 120 | 2 | 7 | 116 | 13 | 1 |
| Ahmedabad .... | 99·4 | 8,812 | 1 | 3 | 88 | 10 | 5 | 71 | 10 | 8 |
| Hyderabad .... | 129·0 | 9,962 | 15 | 11 | 77 | 3 | 8 | 69 | 0 | 10 |
| | 713·3 | 97,978 | 13 | 6 | 137 | 5 | 8 | 131 | 11 | 6 |

